# Mitophagy acts as a safeguard mechanism against human vascular smooth muscle cell apoptosis induced by atherogenic lipids

**DOI:** 10.18632/oncotarget.8936

**Published:** 2016-04-22

**Authors:** Audrey Swiader, Hripsime Nahapetyan, Julien Faccini, Romina D'Angelo, Elodie Mucher, Meyer Elbaz, Patricia Boya, Cécile Vindis

**Affiliations:** ^1^ Inserm, UMR-1048, Institute of Metabolic and Cardiovascular Diseases, Toulouse, France; ^2^ Toulouse University Paul Sabatier, Toulouse, France; ^3^ Department of Cellular and Molecular Biology, Centro de Investigaciones Biológicas, CSIC, Madrid, Spain

**Keywords:** human vascular smooth muscle cell, mitophagy, apoptosis, oxidized lipoproteins, atherosclerosis

## Abstract

Mitophagy is a critical cellular process that selectively targets damaged mitochondria for autophagosomal degradation both under baseline conditions and in response to stress preventing oxidative damage and cell death. Recent studies have linked alterations in mitochondria function and reduced autophagy with the development of age-related pathologies. However, the significance of mitochondrial autophagy in vessel wall in response to atherogenic lipid stressors is not known. In the present study, we investigated the role of mitophagy on human vascular smooth muscle cells (VSMC) apoptosis induced by oxidized low-density lipoproteins (LDL). We reported for the first time that the engulfment of defective mitochondria by autophagosomes occurred in human VSMC in response to oxidized LDL. The molecular mechanism mediating mitophagy in human VSMC involved dynamin-related protein 1 (Drp1)-mediated mitochondrial fission, accumulation of PTEN-induced putative kinase 1 (PINK1) and the recruitment of the E3 ubiquitin ligase Parkin to mitochondria. Likewise, we found increased voltage-dependent anion channel 1 (VDAC1) and mitofusin 2 (Mnf2) mitochondrial proteins ubiquitination and LC3 association to mitochondria. Using flow cytometry in the presence of lysosomal inhibitors, we showed that PINK1 and Parkin silencing impaired mitophagy flux and enhanced oxidized LDL-induced VSMC apoptosis. In addition, overexpression of PINK1 and Parkin were protective by limiting cell death. Moreover, reduced Bax levels found in VSMC-overexpressing Parkin indicated cross talk among mitophagy and mitochondrial apoptotic signalling pathways. Altogether these data demonstrate that mitophagy is a safeguard mechanism against human VSMC apoptosis induced by atherogenic stressors and highlight mitophagy as a potential target to stabilize atherosclerotic plaque.

## INTRODUCTION

Atherosclerosis involves the build-up of fibrous and fatty deposits called plaque inside the arteries. Most of the vulnerable plaques are associated with the presence of highly inflammatory cell content and a large necrotic core covered by a thin “fibrous cap” characterized by few smooth muscle cells (SMC) and less extracellular matrix [1]. Vulnerable plaques are also typified by the accumulation of apoptotic cells and defective phagocytic clearance (efferocytosis), resulting in the lipid-filled necrotic core [2]. The apoptosis of SMC increases as atherosclerotic plaques develop and is sufficient to induce features of plaque vulnerability such as increased necrotic core and plaque inflammation [3]. Besides apoptosis, emerging evidence supports that the induction of vascular cells autophagy may play an important role in the cellular response to various important atherogenic stressors such as oxidized lipids and lipoproteins [4]. We previously showed that oxidized LDL and oxidized lipids induced both apoptotic endoplasmic reticulum (ER) stress and autophagy in human vascular cells and macrophages [5]. Our work has also demonstrated that vascular cells silenced for Beclin-1, a central autophagy regulator protein, exhibited less PS (phosphatidylserine) externalization and uptake by phagocytic macrophages [5, 6]. Moreover, it has been shown that dying SMC in the fibrous cap of advanced human plaques exhibit ubiquitinated inclusions in their cytoplasm evocative of defective autophagy [7]. Recently, the deletion of the essential autophagy gene Atg7 in murine VSMC showed accelerated senescence but also promoted ligation-induced neointima formation thus implying autophagy in the control of VSMC phenotype and proliferation [8]. Overall, VSMC autophagy plays a critical role in physiological and pathological conditions.

However autophagy is not only a non selective process whereby autophagosomes engulf cytosolic material [9]; it is now clear that autophagy specifically targets bacteria, proteins aggregates, and organelles such as mitochondria [10]. Thus, selective autophagy could have different role to degrade specific biological or pathological substrates. Mitophagy, the selective degradation of mitochondria by autophagy [11] can occur in specific developmental processes such as the maturation of erythrocytes [12] but also following pathological mitochondrial damage to eliminate damaged mitochondria and prevent cell death [13]. Indeed, during the early stages of apoptosis, alterations in mitochondrial morphology (fragmentation and remodelling) and function (decline in mitochondrial reduction potential and increase in radical production) are observed, all these events are known as a prerequisite for the initiation of mitophagy [14, 15]. Therefore, the selective elimination of dysfunctional mitochondria reduces the release of cytochrome c and other pro-apoptotic factors into the cytosol which activates downstream cell death pathways [16].

In mammalian cells, there are two different mechanisms for damaged mitochondria to be recognized by the autophagic machinery. The most well established mechanism involves the PTEN-induced putative kinase 1 (*PINK1*)/E3 ubiquitin ligase Parkin pathway and autophagy adaptors proteins such as p62/SQSTM1 [17, 18]. The second mechanism involves specific mitochondrial receptors, such as BNIP3, NIX and FUNDC1. Those proteins function as autophagy receptors by directly binding to autophagosomal membranes through their LC3 interacting region/LIR. Recently, a study has demonstrated that the externalized cardiolipin can also bind to LC3 on the autophagosome [17, 19].

Accumulating evidences have linked impaired mitophagy with age-related pathologies such as Parkinson's disease or heart failure thus suggesting a beneficial role of mitophagy. However, although the relationship between mitophagy and Parkinson's or cardiac diseases is extensively studied [14, 17], the significance of mitochondrial autophagy in vessel wall during atherogenic stress-induced apoptosis is not known. We hypothesized that mitophagy by removing damaged mitochondria could be a safeguard mechanism to prevent vascular cell death and contribute to atherosclerotic plaque stabilization.

Therefore, in the present study we sought to address: i) whether the engulfment of defective mitochondria by autophagosomes occurs in human vascular SMC (VSMC) in response to oxidized LDL-induced apoptosis if any; ii) what are the associated signalling pathways underlying oxidized LDL-activated mitophagy and iii) whether the modulation of mitophagy has an effect on oxidized LDL-induced human VSMC apoptosis.

Our results reported for the first time that a selective removal of damaged mitochondria through autophagy takes place in human VSMC exposed to oxidized LDL. We also provided evidence that mitophagy is dependent on the PINK1/Parkin pathway and acts to safeguard human VSMC against apoptosis induced by atherogenic stressors.

## RESULTS

### Mitochondrial depolarization, dysfunction and fission are triggered by oxidized LDL in human VSMC

**Figure 1:**
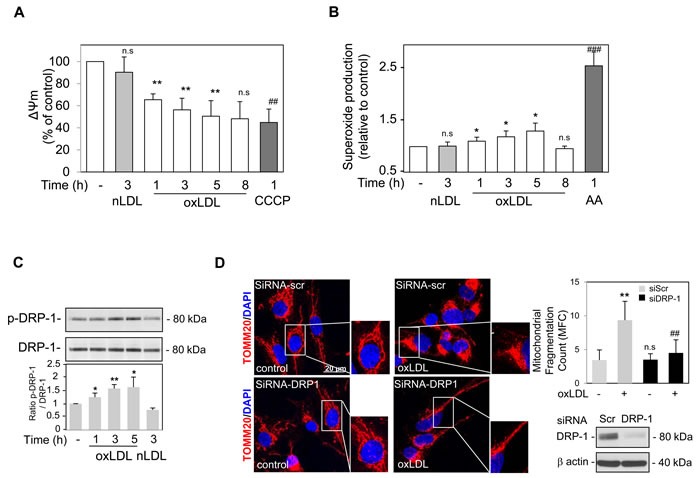
Oxidized LDL induced mitochondrial depolarization, dysfunction and Drp-1 dependent fission in human VSMC **A.** Time course analysis of the mitochondrial membrane potential (ΔΨm). Human VSMC were labelled with the JC-1 dye and stimulated with oxidized or native LDL (respectively oxLDL and nLDL) (200 μg ApoB/mL), at the indicated times or with CCCP (20 μM). JC-1 exhibits potential-dependent accumulation in mitochondria. At low membrane potentials, JC-1 exists as a monomer and produces a green fluorescence (emission at 520 nm). At high membrane potentials or concentrations, JC-1 forms aggregates and produces a red fluorescence (emission at 590 nm). The ratio 590/520 is indicative of mitochondrial membrane depolarization. The data are expressed as mean ± SEM of 5 separate experiments, ** *P* < 0.01 and ## *P* < 0.01 indicate significance, n.s indicates no significance. **B.** Mitochondrial superoxide formation was detected using MitoSOX Red dye (excitation/emission at λ = 510 nm/580 nm) in human VSMC treated with oxidized or native LDL (respectively oxLDL and nLDL) (200 μg ApoB/mL) or Antimycin A (AA) (10 μM) at the indicated times and the relative fluorescence intensity is quantified. The data are expressed as mean ± SEM of 5 separate experiments, * *P* < 0.05, ** *P* < 0.01 and ## *P* < 0.01 indicate significance, n.s indicates no significance. **C.** Immunoblot analysis of the fission protein Drp-1 following oxidized or native LDL treatment. Human VSMC were stimulated with oxidized (oxLDL) or native LDL (nLDL) (200 μg ApoB/mL) at the indicated times and Western blot experiments were performed on total protein extracts using anti-phosphorylated Drp-1(Ser616) antibody and total Drp-1 expression was used as loading control. Blots are representative of 4 independent experiments. The graph represents values of phosphorylated Drp-1(Ser616) band intensity after normalization for total Drp-1 band intensity by densitometry, * *P* < 0.05 and ** *P* < 0.01 indicate significance. **D.** Representative images of mitochondrial fragmentation/fission. Reversal of the mitochondrial fragmentation in human VSMC was achieved using siRNA mediated knockdown of Drp-1 expression. Images are representative of human VSMC treated with oxidized LDL (200 μg ApoB/mL) for 5 h, undergoing siRNA Drp-1 or siRNA scramble transfection. Mitochondria were stained using an antibody against the outer mitochondrial membrane-localized protein TOMM20 (red). Nuclei (blue) were stained with DAPI (4ʹ,6-diamidino-2-phenylindole). The graph represents the quantification of the MFC and shows a significant reduction in oxidized LDL stimulated cells transfected with siRNA Drp-1. Data are expressed as mean ± SEM of 3 separate experiments, ** *P* < 0.01 and ## *P* < 0.01 indicate significance, n.s indicates no significance.

Mitochondrial membrane potential (ΔΨm) is the driving force for mitochondrial ATP synthesis. Mitochondrial depolarization indicates impaired mitochondrial function and is a prerequisite for the initiation of mitophagy [14]. Following oxidized LDL treatment at a concentration previously shown to induce both apoptotic and autophagy signaling [5, 6], the ΔΨm measured in human VSMC was significantly decreased, as indicated by a decrease in the ratio of red to green JC-1 fluorescence intensity (Figure 1A). To study the effects of oxidized LDL on mitochondrial function, we used a mitochondrial targeted fluorescent superoxide sensor. Human VSMC treated with oxidized LDL displayed an increased mitochondrial superoxide generation as measured by MitoSOX fluorescence, indicating that oxidized LDL promoted an increase in mitochondrial ROS (Figure 1B). Native LDL which did not induce mitochondrial apoptotic signalling in vascular cells as we previously showed [20] had no effects on ΔΨm and mitochondrial ROS generation. Because mitochondrial fission plays a key role in segregating mitochondria from the network and is necessary to trigger mitophagy, we examined whether mitochondrial dysfunction after oxidized LDL treatment was associated with changes in mitochondria morphology [23]. Since the activity of the cytoplasmic dynamin-related protein 1 (Drp-1), the primary regulator of mitochondrial fission, is regulated by phosphorylation we studied its phosphorylation at Ser616, which enhances Drp-1 activity. As shown in Figure 1C when compared to untreated VSMC, we found an increase in the Drp-1 Ser616 phosphorylation following oxidized LDL stimulation confirming its activation. We then assessed whether the mitochondrial fragmentation observed in VSMC treated with oxidized LDL could be reversed by either using siRNA-mediated knockdown of Drp-1 or the inhibition of Drp-1 with the small molecule Drp-1 inhibitor Mdivi-1 [24]. Silencing of Drp-1 by specific siRNA (Figure 1D) and the inhibitor Mdivi-1 (Supplemental Figure S-I) markedly inhibited the mitochondrial fragmentation in oxidized LDL treated cells as shown in the representative images of VSMC stained with antibodies against the outer mitochondrial membrane-localized protein TOMM20 and by quantification of the mitochondrial fragmentation count (MFC). Mitochondria appeared to be clustered around the nucleus and smaller in length, loss of this mitochondrial network generated a globular mitochondrial structure indicative of mitochondrial fragmentation. The breakdown of the mitochondrial network after oxidized LDL treatment was also illustrated by ultrastructural analysis showing smaller and spherical mitochondria in human VSMC (Supplemental Figure S-II).

### PINK1 and Parkin are recruited to the damaged mitochondria upon oxidized LDL exposure in human VSMC

**Figure 2:**
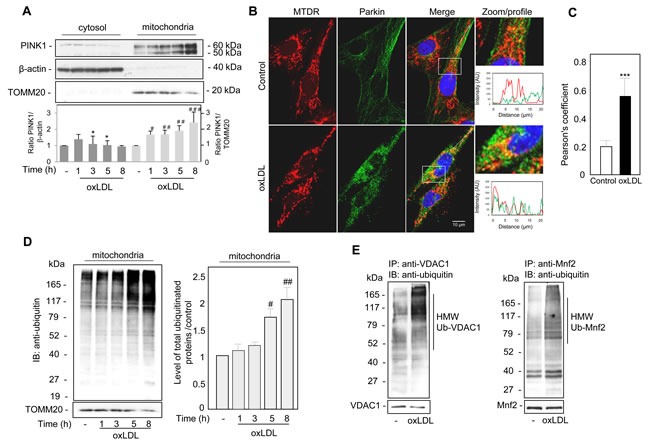
Oxidized LDL induced the recruitment of PINK1 and Parkin to the damaged mitochondria and increased mitochondrial ubiquitinylated proteins in human VSMC **A.** Immunoblot analysis of the 60-kDa full-length and the 50-kDa cleaved endogenous PINK1 expression in cytosolic and mitochondrial fractions of human VSMC treated with oxidized LDL (200 μg ApoB/mL) at the indicated times. The cellular fractions were probed for PINK1 and the cytosol and mitochondrial markers β-actin and TOMM20, respectively. Blots are representative of 4 independent experiments. The graph represents values (means ± SEM) of PINK1 band intensity after normalization for β-actin and TOMM20 by densitometry, * *P* < 0.05, # *P* < 0.05, ## *P* < 0.01 and ### *P* < 0.001 indicate significance. **B.** Representative images of Parkin translocation to the mitochondria, human VSMC were stimulated with oxidized LDL (200 μg ApoB/mL) for 5 h. Mitochondria were immunostained with Mito Tracker Deep Red (MTDR, red) and Parkin antibody. **C.** The Pearson coefficient indexes between Parkin and MTDR fluorescence intensity were determined in regions of interest (ROI) for 10 or more cells in 3 independent experiments. The values are the means ± SEM; ** *P* < 0.01 indicates significance. **D.** Immunoblot analysis of total ubiquitinated proteins in mitochondrial fractions of human VSMC treated with oxidized LDL (200 μg ApoB/mL) at the indicated times. The cellular fractions were probed for ubiquitin and immunoblots are representative of 3 independent experiments. The graph represents the densitometric analysis of mitochondrial ubiquitinated protein intensity relative to untreated control, # *P* < 0.05 and ## *P* < 0.01 and indicate significance. **E.** Immunoprecipitations of VDAC1 and Mnf2 from human VSMC confirm ubiquitylation of both proteins. Oxidized LDL (200 μg ApoB/mL, 5 h) treatment increases high molecular weight (HMW) ubiquitylated species of VDAC1 and Mnf2. Using specific antibodies VDAC1 and Mnf2 were immunoprecipitated from lysates, western blotted and probed with anti-ubiquitin, anti-VDAC1 and anti-Mnf2 antibodies.

The regulation of mitophagy could involve *PINK1* a mitochondrial serine/threonine-protein kinase, which is constitutively imported into the mitochondria. Indeed, upon loss of ΔΨm, PINK1 accumulates on the outer mitochondrial membrane (OMM). We investigated the subcellular localization of endogenous PINK1 in human VSMC after oxidized LDL stimulation by analyzing the distribution of PINK1 in the cytosol and the mitochondria using a biochemical approach. In fractionation experiments, we found that time course oxidized LDL treatment promoted the gradual accumulation of both the 60-kDa full-length and the 50-kDa cleaved endogenous PINK1 in the mitochondria-rich fraction (Figure 2A). PINK1 acts as an upstream factor for the E3 ubiquitin ligase Parkin and is essential both for the activation of E3 Parkin activity and for recruiting Parkin onto depolarized mitochondria. Confocal microscopy (Figure 2B) showed that under steady-state conditions, endogenous Parkin was diffusely localized throughout the cytosol [25] and revealed that oxidized LDL exposure triggered the redistribution of Parkin to the mitochondria. The green Parkin fluorescence intensity profile overlaps with the mitochondria-selective probe Mito Tracker Deep Red (MTDR) staining under oxidized LDL treatment, showing colocalization events between Parkin and mitochondria. The significantly higher degree of colocalization between Parkin and mitochondria was then quantified by Pearson's correlation coefficient, representing the linear relationship of the signal intensity from the green and red channels (Figure 2C). In parallel with Parkin mitochondrial location, we found that a marked increase in the abundance of ubiquitinated proteins occurred in the mitochondria-rich fraction of oxidized LDL-treated human VSMC (Figure 2D). VDAC1 (voltage-dependent anion channel 1) and Mnf2 (mitofusin 2) have been identified as mitochondrial targets for Parkin-mediated poly-ubiquitylation and mitophagy [26, 27]. We then determined whether endogenous VDAC1 and Mfn2 becomes ubiquitinated, to this end, we analyzed denatured VDAC1 and Mfn2 immunoprecipitates (IP) from control and oxidized LDL-treated human VSMC. The data showed that oxidized LDL treatment induced high molecular weight (HMW) species of VDAC1 and Mfn2 that cross-reacted with anti-ubiquitin antibodies (Figure 2E).

### Oxidized LDL-promoted dysfunctional mitochondria are targeted to the autophagy machinery in human VSMC

**Figure 3:**
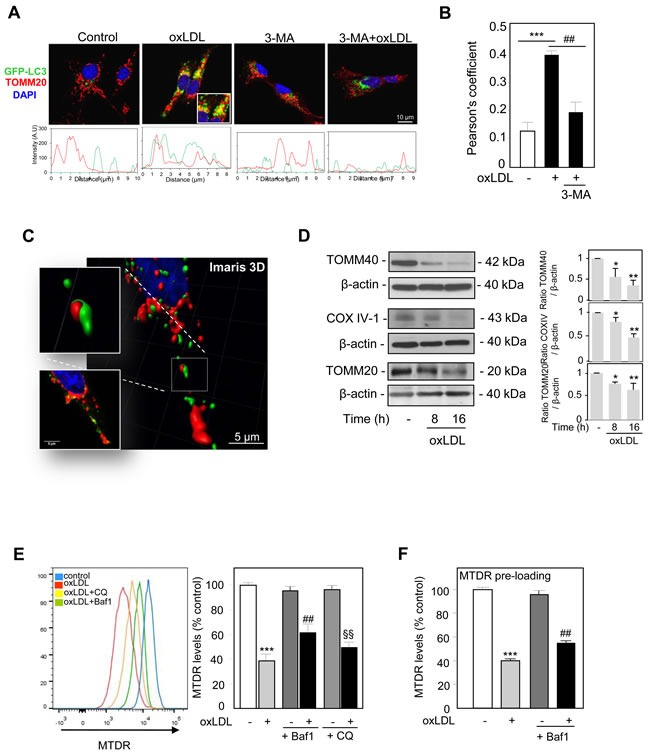
Mitophagy monitoring in oxidized LDL-stimulated human VSMC **A.** Analysis of mitochondria engulfment by autophagosomes using dual fluorescence. Human VSMC were transfected with GFP-LC3 (green) and the colocalization of GFP-LC3 with the mitochondria marker TOMM20 (red) was determined following oxidized LDL (200 μg ApoB/mL, 8h) treatment with or without 3-MA. **B.** The Pearson coefficient indexes between GFP-LC3 intensity and TOMM20 intensity were determined in regions of interest (ROI) for 5 or more cells in 3 independent experiments. The values are the means ± SEM; *** *P* < 0.001 and ## *P* < 0.01 indicate significance. **C.** Three-dimensional reconstruction of confocal fluorescence microscopy z-stack slices showed that mitochondria are present within autophagosomes following oxidized LDL treatment in human VSMC. **D.** Immunoblot analysis of the mitochondrial proteins TOMM40, COX IV-1 and TOMM20 in human VSMC treated with oxidized LDL (200 μg ApoB/mL) for the indicated times. The blots are representative of 3 independent experiments. The graph represents values (means ± SEM) of TOMM40, COX IV and TOMM20 band intensities after normalization for β-actin by densitometry. ** *P* < 0.01 and * *P* < 0.05 indicate significance. **E.** Flow cytometry analysis of mitophagy in human VSMC after oxidized LDL treatment. Cells were incubated with oxidized LDL (200 μg ApoB/mL) for 8 h and treated with or without the lysosomal inhibitors Baf1 (10 nM) and chloroquine CQ (10μM) 3 h before analysis to block lysosomal degradation. Human VSMC were then stained with MTDR for flow cytometry analysis. The data are expressed as mean ± SEM of 4 separate experiments, *** *P* < 0.001, ## *P* < 0.01 and §§ *P* < 0.01 indicate significance. **F.** Additional aspects of mitophagy analysis. The preloading of MTDR shows a mitophagy dependent reduction in fluorescence levels. Human VSMC were preincubated with MTDR for 20 min to load mitochondria, washed and then treated with oxidized LDL (200 μg ApoB/mL) for 8 h and treated with or without Baf1 (10 nM) 3 h before flow cytometry analysis to block lysosomal degradation. The data are expressed as mean ± SEM of 4 separate experiments, *** *P* < 0.01 and ## *P* < 0.01 indicate significance.

Parkin ubiquitinates mitochondrial proteins, which serve as signals to recruit the autophagy machinery around damage mitochondria. To determine whether damaged mitochondria were degraded by the autophagy-lysosome pathway, we assessed the colocalization between GFP-LC3 positive autophagosomes and TOMM20-labeled mitochondria upon oxidized LDL exposure. A common method for detecting autophagy is by monitoring the conversion of GFP-tagged microtubule-associated protein 1 light chain 3 (MAP-LC3), from a diffuse cytosolic form (LC3-I) to a lipidated form (LC3-II) that forms part of the autophagic membrane and can be visualized as puncta accumulation by fluorescence microscopy. Using GFP-tagged LC3 to monitor the formation of autophagosomes, we found that oxidized LDL strongly induced the formation of GFP-LC3 puncta, along with mitochondrial clustering (stained by TOMM20, red). The GFP-LC3 (green) fluorescent-intensity profile overlaps with the mitochondria marker TOMM20 (red) staining under oxidized LDL treatment, showing colocalization events between autophagosomes and mitochondria which are prevented by the inhibitor 3-MA (Figure 3A). The significantly higher degree of colocalization between GFP-LC3 and TOMM20 was then quantified by Pearson's correlation coefficient, representing the linear relationship of the signal intensity from the green and red channels (Figure 3B). Three-dimensional reconstruction of confocal fluorescence microscopy z-stack slices was generated using Imaris 3D software. The raw, unprocessed image of a region of a cell is shown in Figure 3C. After projection and thresholding, it can be seen that mitochondria (red) were present within autophagosomes (green) when human VSMC were stimulated with oxidized LDL. Autophagy vesicles and mitochondria engulfment by autophagosome after oxidized LDL treatment were also shown by electron microscopy analysis (Supplemental Figure S-III). These findings were supported by western blot analysis; oxidized LDL treatment decreased the levels of outer and inner mitochondrial proteins including TOMM40, COX IV-1 and TOMM20 (Figure 3D). In addition we corroborated these findings with the quantitative measure of mitophagy in our cellular model. For this purpose we used a recently described and new quantitative method to measure mitophagic flux using flow cytometry in the presence of lysosomal inhibitors [22]. As shown, oxidized LDL treatment at the dose of 200 μg ApoB/mL (Figure 3E) resulted in decreased MTDR staining that was blocked by the lysosomal inhibitors Bafilomycin A1 (Baf1) and chloroquine (CQ). Lower concentrations of oxidized LDL or native LDL didn't display significant decreased MTDR staining (Supplemental Figure S-IV), in comparison to carbonyl cyanide m-chlorophenyl hydrazone (CCCP) incubation, a classical inducer of mitophagy (Supplemental Figure S-IV). Interestingly, the decrease in the MTDR fluorescence levels after oxidized LDL treatment was also observed when MTDR was incubated before the treatments, indicating that the fluorescence intensity reduction was not due to reduced loading of the probe following oxidized LDL treatment (Figure 3F). Evidences for oxidized LDL-induced mitophagy activity were also obtained by measuring the appearance of the lipidated form of LC3 (LC3-II) and Beclin 1 expression increase after oxidized LDL treatment (Supplemental Figure S-VA, B). The autophagy flux increase was demonstrated in cells stimulated with oxidized LDL in the presence of Bafilomycin A1[28] (Supplemental Figure S-VC).

### Mitophagy displays a protective role against human VSMC apoptosis induced by oxidized LDL

**Figure 4:**
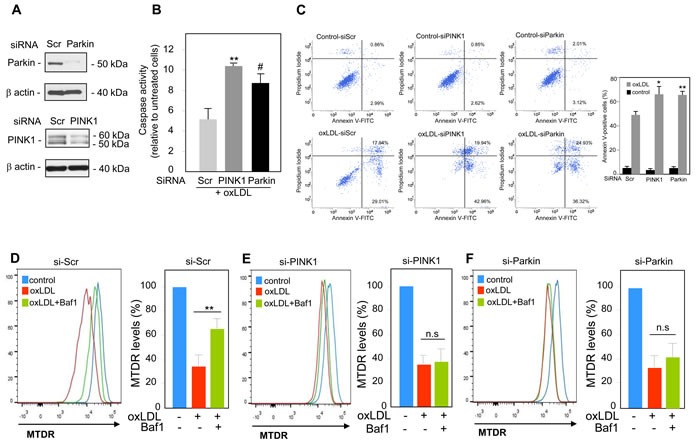
PINK1 and Parkin knockdown enhances human VSMC apoptosis mediated by oxidized LDL Analysis of oxidized LDL-induced apoptosis in human VSMC transfected with scrambled (si-Scr, 100 nM), (si-Parkin, 100 nM) or (si-PINK1 100 nM), siRNA for 24 h and then incubated with oxidized LDL (200 μg ApoB/mL) for 16 h. **A.** shows the western-blot analysis of PINK1 and Parkin expressions in human VSMC after siRNA transfection. **B.** Assessment of caspases activity in PINK1 and Parkin-deficient human VSMC. Whole cell detection of caspase activity in apoptotic or caspase-positive cells was performed using the membrane-permeant, fluorescent inhibitor-based FLICA caspase probes as described under “Materials and Methods”. The data are expressed as mean ± SEM of 3 separate experiments, ** *P* < 0.01 and # *P* < 0.05 indicate significance. **C.** Apoptosis of control, PINK1 and Parkin-deficient human VSMC stimulated with oxidized LDL (200 μg ApoB/mL) for 16 h was determined by Annexin V/PI staining followed by flow cytometry analysis. The graph represents the quantitative analysis of the percentage of Annexin V-FITC positive cells. The data are expressed as mean ± SEM of 5 separate experiments, * *P* < 0.05 and ** *P* < 0.01 indicate significance. Mitophagy assessment in **D.** human VSMC transfected with scrambled siRNA (si-Scr); **E.** human VSMC transfected with PINK1 siRNA (si-PINK1) and **F.** human VSMC transfected with Parkin siRNA (si-Parkin), and incubated with oxidized LDL (200 μg ApoB/mL) for 8 h. Cells were treated with or without Baf1 (10 nM) 3 h before analysis to block lysosomal degradation. Human VSMC were then stained with MTDR for flow cytometry analysis. The data are expressed as mean ± SEM of 6 separate experiments, ** *P* < 0.01 indicates significance.

**Figure 5:**
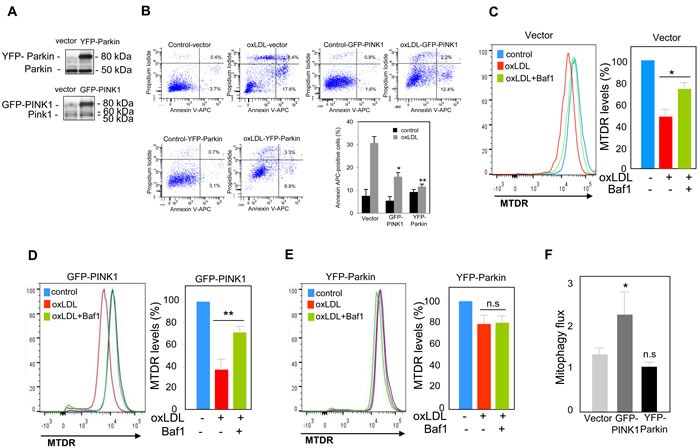
PINK1 and Parkin overexpression protects human VSMC against oxidized LDL-induced apoptosis **A.**, **B.** Analysis of oxidized LDL-induced apoptosis in cells transfected with EGFP vector, YFP-Parkin vector or GFP-PINK1 vector. **A.** Immunoblot analysis of PINK1 and Parkin overexpression in human VSMC after 24 h of transfection. **B.** Apoptosis of control, PINK1 and Parkin-overexpressing VSMC stimulated with oxidized LDL (200 μg ApoB/mL) for 16 h was determined by Annexin V-APC/PI staining followed by flow cytometry analysis. The graph represents the quantitative analysis of the percentage of Annexin V-APC positive cells. The data are expressed as mean ± SEM of 5 separate experiments, * *P* < 0.05 and ** *P* < 0.01 indicate significance. Mitophagy assessment in **C.** human VSMC transfected with EGFP vector; **D.** human VSMC transfected with GFP-PINK1 vector and **E.** human VSMC transfected with YFP-Parkin vector, and incubated with oxidized LDL (200 μg ApoB/mL) for 8 h. Cells were treated with or without Baf1 (10 nM) 3 h before analysis to block lysosomal degradation. Cells were then stained with MTDR for flow cytometry analysis and the MTDR fluorescence was determined in the GFP-positive population. The data are expressed as mean ± SEM of 6 separate experiments, * *P* < 0.05 and ** *P* < 0.01 indicate significance, n.s (non significant). **F.** Mitophagy flux determination in human VSMC transfected with EGFP vector or GFP-PINK1 or YFP-Parkin, and incubated with oxidized LDL (200 μg ApoB/mL) for 8 h in the presence of Baf1 (10 nM). The data are expressed as mean ± SEM of 6 separate experiments. *P < 0.05 indicates significance, n.s indicates no significance.

We next investigated the functional role of mitophagy triggered by oxidized LDL in human VSMC. The PINK1-Parkin pathway has been shown to have cytoprotective and anti-apoptotic activities through the clearance of damaged mitochondria [13]. In order to evaluate the outcomes associated with perturbations in the PINK1-Parkin pathway, we generated human VSMC cells that either over-express or exhibit knock-down expression of endogenous PINK1 or Parkin. We used a siRNA strategy to knock-down PINK1 or Parkin expression in human VSMC (Figure 4A). The consequences on cell fate were illustrated by the significant increase in the caspase activity (Figure 4B) and in the number of apoptotic cells (Figure 4C) both in PINK1- and Parkin-deficient human VSMC following oxidized LDL treatment compared to scramble siRNA transfected cells. We then asked whether the increase of cell death was a consequence of impaired mitophagy. The decrease of MTDR signal measured in oxidized LDL-treated cells was not significantly prevented when the PINK1- or Parkin-deficient cells were treated with oxidized LDL and Baf1 confirming that mitophagy triggered by oxidized LDL was impaired and that PINK1 and Parkin expression was necessary for mitophagy induction (Figure 4D–4F). Altogether these data demonstrated that mitophagy was a safeguard mechanism against oxidized LDL-induced human VSMC apoptosis. Therefore, we investigated whether PINK1 or Parkin overexpression in human VSMC could confer a better protection to oxidized LDL-induced apoptosis. PINK1- or Parkin-overexpressing human VSMC (Figure 5A) exhibited enhanced cell survival towards oxidized LDL-induced apoptosis as demonstrated by a markedly decrease in the number of apoptotic cells (Figure 5B). This result raised the question whether this anti-apoptotic effect was a consequence of boosted mitophagy. As shown in Figure 5C and 5D, the decrease of MTDR signal induced by oxidized LDL treatment, was significantly prevented when the control human VSMC or PINK1-overexpressing human VSMC were treated with Baf1 confirming the degradation of mitochondria by autophagy. Unexpectedly, following oxidized LDL stimulation Parkin-overexpressing human VSMC didn't display a significant loss of MTDR signal compared to control human VSMC (Figure 5E). We then measured the mitophagy flux as the ratio of the MTDR fluorescence in the presence of Baf1 to that in the absence of Baf1, normalized to the corresponding value in control cells. The mitophagy flux in PINK1-overexpressing human VSMC was significantly increased compared to control cells confirming a boosted mitophagy but not in Parkin-overexpressing cells (Figure 5F).

Off target effects of Parkin plasmid overexpression could be ruled out because forced expression of Parkin in Hela cells (Hela-Parkin) prevented oxidized LDL-induced cell death without inducing mitophagy flux compared to wild type Hela cells that express mutated Parkin protein [29] (Supplemental Figure S-VIA, C).

**Figure 6:**
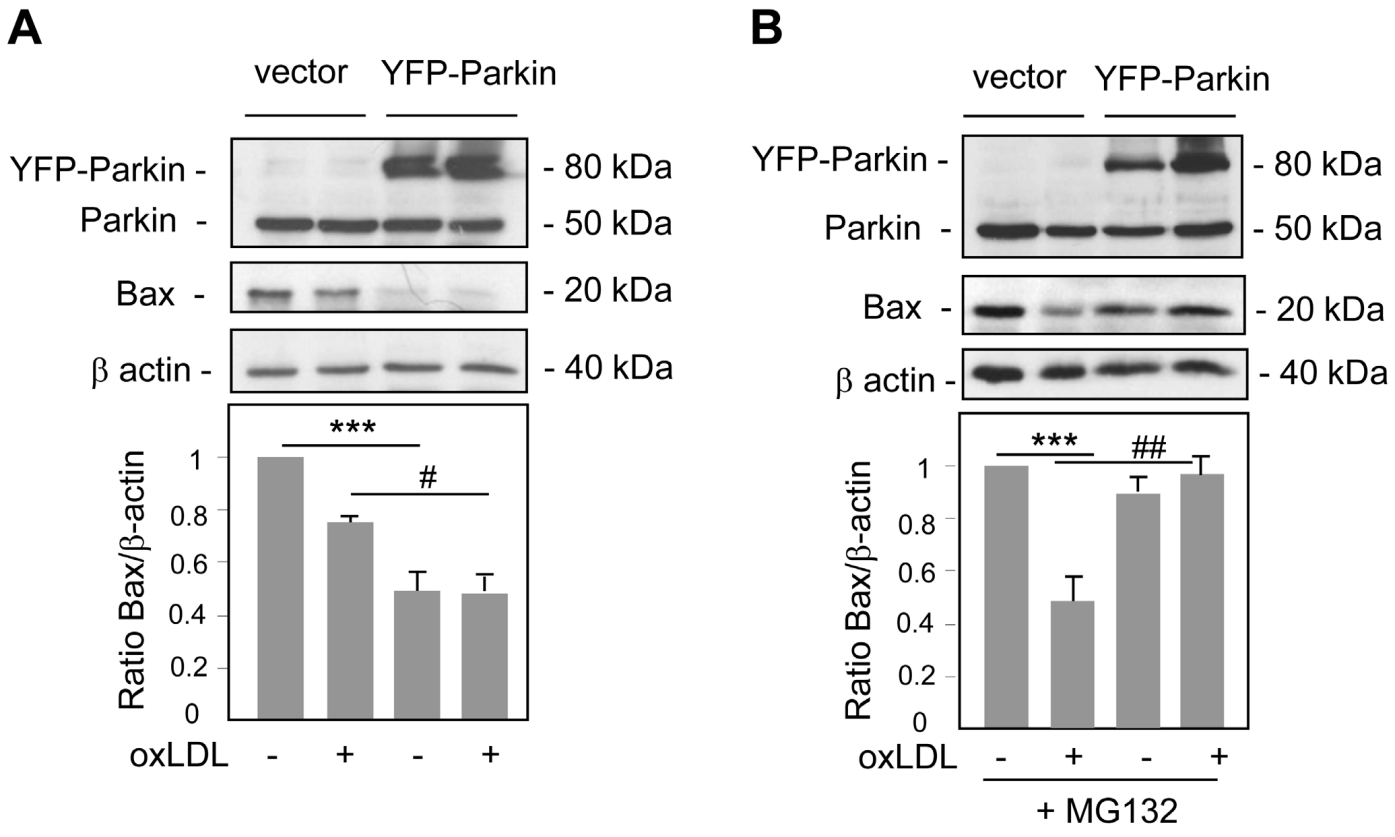
Parkin overexpression decreases Bax levels **A.** Immunoblot analysis of Bax and Parkin expression in human VSMC transfected with YFP-Parkin or control vector, and incubated with oxidized LDL (200 μg ApoB/mL) for 8 h. Blots are representative of 3 independent experiments. The graph represents values (means ± SEM) of Bax band intensity after normalization for β-actin by densitometry. *** *P* < 0.001 and # *P* < 0.05 indicate significance. **B.** Immunoblot analysis of Bax and Parkin expression in human VSMC transfected with YFP-Parkin or control vector, pretreated with a proteasome inhibitor MG132 (10 μM) and incubated with oxidized LDL (200 μg ApoB/mL) for 8 h. Blots are representative of 3 independent experiments. The graph represents values (means ± SEM) of Bax band intensity after normalization for β-actin by densitometry. *** *P* < 0.001 and ## P < 0.01 indicate significance.

Altogether these results suggested the existence of two pathways of Parkin function to promote cell survival one requiring PINK1 for the mitophagy process and the other involving regulation of mitochondrial apoptotic pathway. Interestingly, prior works have demonstrated that Parkin regulated the proapoptotic function and the level of Bax in multiple Parkin-overexpressing cell culture systems [30]. To address this issue we analyzed the expression of Bax in Parkin-overexpressing human VSMC treated with oxidized LDL. As shown in Figure 6A the levels of Bax in whole cell lysates were significantly reduced in Parkin-overexpressing cells compared to control cells (also seen in Hela-Parkin cells, Supplemental Figure S-VIB) which could explain in part its antiapoptotic role. Furthermore, the use of proteasomal inhibitor increased Bax expression in Parkin-overexpressing human VSMC (Figure 6B) indicating that Parkin overexpression induced Bax degradation *via* the Ub-proteasome system.

Finally, our data have provided clear evidence that mitophagy played a critical role in the protection against human VSMC apoptosis induced by oxidized LDL.

## DISCUSSION

VSMC apoptosis plays a critical role during the progression of atherosclerosis and is a common feature of high-risk/vulnerable atherosclerotic plaques. The goal of the present study was to investigate the occurrence of mitophagy and its functional role in VSMC challenged with atherogenic factors known to activate apoptosis. To our knowledge this is the first evidence showing that the selective removal of damaged mitochondria through autophagy takes place in human VSMC exposed to oxidized LDL. Using loss- or gain-of-expression approaches we reported that mitophagy had critical consequences on cell fate by enhancing apoptosis or by favouring cell survival. First, we provided convincing evidence that the molecular mechanisms mediating mitophagy in human VSMC involved: i/ Drp1-induced mitochondrial fission as shown by its silencing and phosphorylation on Ser616 and, ii/ the accumulation of PINK1 upon loss of the ΔΨm and the recruitment of the E3 ubiquitin ligase Parkin from the cytosol to the mitochondrial membrane. Our data are in agreement with studies showing that mitochondrial fission must occur prior to mitophagy [23], one explanation could be that fission produced smaller mitochondrial fragments that can more easily be engulfed by autophagosomes [13]. Moreover, the inhibition of mitochondrial fission resulted in reduction of the number of mitochondria-containing autophagosomes [31]. In addition our results are supported by studies that have found that upon collapse of the ΔΨm, PINK1 accumulates and stabilizes on the OMM, which allows the recruitment and the activation of Parkin [25]. Upon translocation it has been reported that Parkin ubiquitinates a broad range of mitochondrial membrane proteins [27] which permits mitochondria recognition for autophagic degradation. Parkin-dependent mitochondrial ubiquitination subsequently results in the recruitment of adapter proteins and interaction with the autophagosome formation site [14]. Consistent with this, we found in human VSMC challenged with oxidized LDL a translocation of Parkin to the mitochondria, an increase in the ubiquitination of mitochondrial proteins and the association of LC3 with mitochondria. Moreover, we showed enhanced Beclin 1 levels and appearance of the lipidated form of LC3, which contributed to the formation and elongation of autophagosomes. Interestingly, the quantification of mitophagy using a new quantitative method to measure mitophagic flux let us to demonstrate that oxidized LDL was a potent inducer of mitophagy in human VSMC. Basal autophagy is a necessary process for proper vascular function and accumulating evidence indicates that autophagy is also stimulated by stress-related stimuli in the vascular wall [32]. However, although mitophagy is important in mitochondrial control quality both under baseline conditions and in response to stress, its functional role in the context of vascular pathology is currently unknown. Consistent with the concept that successful mitophagy protects against oxidative stress and from the release of proteins that participate in cell death pathways, we showed that mitophagy safeguarded human VSMC against oxidized LDL-induced apoptosis. Interestingly our results strengthen that autophagy and apoptosis in atherogenic situation can be elicited by common upstream regulators, such as impaired cellular organelles. Indeed, dysfunctional mitochondria, formerly recognized as an initial event in cell apoptosis, are also a trigger of autophagy (summary scheme in Supplemental Figure S-VII).

To our knowledge the potential role of PINK1 and Parkin in VSMC has never been investigated, their critical role is now substantiated by our results showing that loss of PINK1 and Parkin enhanced caspase activity and cell death mediated by oxidized LDL thus supporting their anti-apoptotic function. Interestingly, the loss of MTDR levels observed in PINK1 and Parkin-deficient VSMC after oxidized LDL stimulation suggests that distinct mitophagy pathways independent of PINK1 and Parkin could cooperate to regulate mitochondrial homeostasis as it was recently demonstrated [31]. It should be noted that our data are coherent with previous studies in other types of muscle cells. For instance, PINK1 deficient-mice had enhanced oxidative stress and higher degrees of cardiomyocyte apoptosis [33] Kubli and coworkers [34] showed that mice with global deletion of Parkin were more sensitive to myocardial infarction, which was mainly attributed to the impaired mitophagy in cardiomyocytes. In skeletal muscle cells the removal of drosophila PINK1 resulted in muscle degeneration through an apoptotic mechanism [35, 36]. Moreover primary cultures of skeletal muscle derived from Parkin knock-out mice displayed more sensitivity to the toxic effects of β-amyloid [37]. Conversely, over-expressing PINK1 in HL-1 cardiac cells reduced cell death following simulated ischemia reperfusion injury [38]. In addition, Parkin overexpression in adult rat cardiomyocytes conferred a protection against hypoxia-mediated cell death [34]. Although our data clearly demonstrated that mitophagy played an important role in the regulation of VSMC apoptosis, the absence of boosted mitophagy in Parkin-overexpressing cells raised an intriguingly question about the functions of Parkin. The Parkin-dependent reduction in the pro-apoptotic Bax suggested that Parkin plays a role in two distinct mitochondrial pathways to promote cell survival, one requiring PINK1 for initiating mitophagy and another PINK-independent process regulating the mitochondrial apoptotic pathway. Our hypotheses are strengthened by recent reports showing that primary cultured neurons from Parkin knock-out mice accumulated greater levels of Bax at the mitochondria than wild type neurons after apoptotic stimulation [39]. The reduced Bax levels observed in human VSMC-overexpressing Parkin, seems not involved effects on transcription, but rather required Parkin-dependent ubiquitination of Bax and proteasomal degradation [30, 40]. Therefore, we cannot exclude the coexistence and cooperation of both Parkin-dependent regulations of mitochondrial clearance and apoptotic pathways within the same cells. Emerging studies have linked impaired mitophagy to aging and development of age-related cardiac pathologies; however our knowledge regarding mitophagy in the vessel and VSMC therein is limited. We recently reported that autophagy contributed to the generation of engulfment signals required for the phagocytic removal of dying vascular cells exposed to atherogenic factors which was consistent with the intricate link between apoptosis and autophagy [6, 41]. Autophagy has also been involved in cholesterol efflux from foam cells [42] and phenotypic switch of SMC [8]. Moreover in the setting of atherosclerosis progression, it was proposed that autophagy turned deficient thereby resulting in inflammasome hyperactivation through mechanisms that might include generation of ROS and impaired mitophagy [43, 44]. All together, these data illustrated that general autophagy acted as a cellular defense mechanism to protect plaque cells against inflammation, oxidative and metabolic stress.

Here, our current data shed new light on the role of autophagy triggered by atherogenic stressors. Indeed by facilitating the clearance of impaired cellular organelles this selective autophagy triggered by oxidized LDL displayed an additional protective function. Therefore, our findings raise the interest to target selectively mitophagy as a potential target to stabilize atherosclerotic plaque. Indeed, the elimination of damaged mitochondria without enhancing general autophagy would help to minimize off-targets effects induced by the activation of non-selective autophagy. In conclusion, our study provides novel insights in the role of mitophagy in VSMC apoptosis and underlines the relevance of its function in conditions where cell death contributes to disease progression.

## MATERIALS AND METHODS

### Reagents and antibodies

Bafilomycin A1, chloroquine, MTT [3-(4,5-dimethylthiazol-2-yl)-2,5-diphenyltetrazolium bromide], CCCP carbonyl cyanide m-chlorophenyl hydrazone, propidium iodide, 3-Methyladenine were purchased from Sigma-Aldrich (B1793, C6628, C2759, P4170, M2128, respectively). SYTO-13 (S7575) was purchased from Lifetechnologies. Anti-LC3B (2775), anti-Bax (2772), anti-Beclin 1 (3738), anti-SQSTM1/p62 (5114), anti-VDAC1 (4661), anti p-Drp1 (Ser616) (3455), anti-Drp1 (8570), anti-PINK1 (6946), anti-Mnf2 (9482) and anti-ubiquitin (3936) antibodies were from Cell Signaling Technology. Secondary antibodies conjugated to HRP were from Cell Signaling Technology. Anti-β-actin antibody (A2228) was from Sigma-Aldrich, anti-TOMM20 and anti-TOMM40 antibodies were from SantaCruz Biotechnology (sc-11415, sc-11414), anti-Parkin antibody was from Abcam (ab 15954), anti-COX IV subunit I was from Invitrogen (459600).

### Cell culture

Human primary VSMC were obtained from human mesenteric arteries at postmortem examinations. The use of human mesentery from deceased organ donors was approved by the French “Agence de Biomédecine” and the Ethics Committee of the University Hospital of Toulouse, France. All experiments were conformed to the declaration of Helsinki in compliance with French legislation and written informed consent was obtained from relatives for the use of surgery residual tissue for research. Briefly, the arteries were cut longitudinally and small pieces of the media were carefully stripped from the vessel wall and cultured [6]. Within 1-2 weeks, VSMC migrated from the explants; they were capable of being passaged 3 weeks after the first appearance of cells. They were identified as VSMC by their characteristic hill-and-valley growth pattern and immunohistochemistry for VSMC-specific β-actin. The primary cultured human VSMC were used to generate an immortalized cell line by using SV40T antigen, SV40T-expressing human VSMC retain expression of contractile phenotype markers including smooth muscle β-actin and smMHC to passage 10 and higher as we previously described [6]. For all experiments passage 7-17 SV40T-expressing human VSMC culture were used. The cultures were maintained in Dulbecco's modified Eagle's medium (Lifetechnologies) supplemented with 10% fetal calf serum at 37°C in a humidified, 5% CO_2_ / 95% air atmosphere. VSMC were cultured in Dulbecco's modified Eagle's medium without serum when it was mentioned serum starved VSMC. Hela and Hela-Parkin cells were kindly provided by Dr David C. Chan (California Institute of Technology, Howard Hughes Medical Institute Pasadena, CA, USA).

### Plasmids transfection

The plasmids YFP-Parkin (23955), PINK1-GFP (133316), GFP-LC3 (11546) were provided from Addgene. Transient transfection experiments of hVSMC were performed with Lipofectamine-^TM^ reagent (18324-111, Invitrogen) in Opti-MEM medium (31985-047, Lifetechnologies) according to the manufacturer's instructions.

### SiRNA transfection

The selected siRNA specific to human PINK1 were ON-TARGET plus SMART pool siRNA human PINK1 (65018, Dharmacon). The selected siRNA specific to human Parkin were ON-TARGET plus SMART pool siRNA human PARK2 (5071, Dharmacon). The selected siRNA specific to human Drp-1 were ON-TARGET plus SMART pool siRNA human DNM1L (10059, Dharmacon). SiRNAs were transfected using the Hiperfect reagent (301705, Quiagen) according to the manufacturer's recommendations.

### LDL isolation and mild oxidation

LDL from human pooled sera were prepared by ultracentrifugation, dialyzed against PBS containing 100 μM EDTA. LDL were mildly oxidized by UV-C + copper/EDTA (5 μM) as previously reported [20]. Oxidized LDL contained 4.2 to 7.4 nmoles of TBARS (thiobarbituric acid-reactive substances) /μg apoB. Relative electrophoretic mobility (REM) and 2,4,6-trinitrobenzenesulfonic acid (TNBS) reactive amino groups were 1.2-1.3 times and 85-92 % of native LDL, respectively.

### Mitochondrial ROS and membrane potential measurement

Mitochondrial superoxide formation was detected by incubating cells after treatment in the dark with 5 μM MitoSOX Red (M36008, Invitrogen) dye for 30 min and detected at excitation/emission at λ = 510 nm/580 nm) according to the manufacturer's recommendations. To assess mitochondrial membrane potential, the cells were preincubated with 2 μl/mL of JC-1 dye (M34152, Molecular Probes) for 30 min and detected at excitation/emission at λ = 590/610 nm for JC-1 aggregates and excitation/emission at λ = 485/535 nm for monomers according to the manufacturer's recommendations.

### Annexin V/propidium iodide double staining and FACS analysis

Flow cytometry experiments after annexin-V-FITC labeling were performed to evaluate phosphatidylserine externalization, an early event of apoptosis. After specific treatment, cells were collected, resuspended and stained with Annexin V-FITC/ propidium iodide (PI) (human Annexin V-FITC kit, Eurobio, Paris) or Annexin V-APC/ propidium iodide (PI) (human Annexin V-APC kit, Eurobio, Paris) according to the manufacturer's instructions. Cells were analyzed using a LSRFortessa flow cytometer (Becton Dickinson) 20,000 cells were acquired (FACSDiva Software). After appropriate markings for negative and positive populations, the percentage of annexin V^+^/PI^−^ and annexin V^+^/PI^+^ cells was determined and compared with untreated controls.

### Mitochondrial and cytosolic fractionation

Cytosol was separated from mitochondria as previously described [20]. Washed cells were disrupted at 4°C in 20 mmol/L Hepes-KOH buffer pH 7.4, 250 mmol/L sucrose, 10 mmol/L KCl (containing 1 mg/mL bovine serumalbumin, 1 mmol/L EDTA, 2 mmol/L MgCl2, 1 mmol/L DTT, 1 mmol/L PMSF, 10 μg/ml leupeptin, aprotinin and pepstatin) using 25 strokes of the pestle of a tight-fitting ice-cold Dounce homogenizer. After 2 cycles clarification at 2,500 x g for 5 min, supernatant was centrifuged at 12,000 x g for 30 min. The pellet contained the mitochondrial fraction and the supernatant was ultracentrifuged (Beckman Optima) at 100,000 x g for 60 min, to obtain the cytosolic fraction.

### Immunofluorescence and confocal analysis

Cells grown on cover glass slides were washed with PBS and fixed in PBS/4% paraformaldehyde for 10 min, washed and permeabilized with 0.1% TritonX100 for 10 min. After blocking with PBS containing 3% BSA for 30 min, cells were incubated with the primary indicated antibodies for 1 h and revealed with Alexa Fluor-conjugated secondary antibodies (488, 150077 and 568, 175473, Invitrogen) for 1 h. Nuclei were labelled with DAPI and cells were mounted with DAKO fluorescent mounting medium (S3023, DAKO).

The slides were visualized using a Zeiss LSM 780 fluorescence confocal microscope. Images were taken every 0.2 μm and the profile analyses were performed in confocal planes using the Zeiss Zen software. Three-dimensional reconstruction of confocal fluorescence microscopy z-stack slices was generated using Imaris 3D software.

### Mitochondrial network imaging

To quantify structural mitochondrial network fragmentation, cells grown on cover glass slides were stimulated as described, then washed with PBS and fixed in PBS/4% paraformaldehyde for 10 min as described above. After blocking with PBS containing 3% BSA for 30 min, cells were incubated with the primary indicated antibody TOMM20 for 1 h and revealed with Alexa Fluor-conjugated secondary antibody for 1 h. Nuclei were labelled with DAPI and cells were mounted with DAKO fluorescent mounting medium (S3023, DAKO). The slides were visualized using a Zeiss LSM 780 fluorescence confocal microscope. As described in [21], acquired images were background subtracted, filtered (median), thresholded, and binarized to identify mitochondrial segments using Image J. Continous mitochondrial structures were counted with the particle counting subroutine and the number was normalized to the mitochondrial area (in pixels) to obtain the mitochondrial fragmentation count (MFC) for each imaged cell. ≥ n= 25 randomly selected cells for each condition were imaged to calculate the respective MFC values.

### Caspase activity assay

After treatments, cells were incubated with the cell-permeant poly-caspase FLICA™ SR-VAD-FMK reagent (916, ImmunoChemistry Technologies) to quantify apoptosis by measuring intracellular caspase activity *in vitro*. Cells were then analyzed according to the manufacturer's recommendations by 96-well-plate based fluorometry, the sulforhofamine (SR) FLICA reagents have an optimal excitation range of 560 - 570 nm, and emission range from 590 - 600 nm.

### Western blot analysis

After treatments, cells were washed in cold PBS and proteins were extracted in solubilizing buffer (10 mM Tris pH 7.4, 150 mM NaCl, 1% Triton X-100, 1% sodium deoxycholate, 0.1% sodium dodecyl sulfate, 1 mM sodium orthovanadate, 1 mM sodium pyrophosphate, 5 mM sodium fluoride, 1 mM phenylmethylsulfonyl fluoride, 1 μg/mL leupeptin, 1 μg/mL aprotinin) for 30 min on ice. 40 μg of protein cell extracts for Western blot analysis were resolved by SDS-polyacrylamide gel electrophoresis, transferred onto PVDF membranes (Immobilon, IPVH 00010, Millipore). Then membranes were probed with the indicated primary antibodies and revealed with the secondary antibodies coupled to horseradish peroxidase using the ECL chemoluminescence kit (RPN21016, Amersham). Membranes were then stripped and reprobed with anti-β-actin antibody to control equal loading of proteins.

### Immunoprecipitation

Immunoprecipitations were carried out by using protein A- or protein G-coated sepharose beads (GE Healthcare, Chalfont St. Giles, UK) following manufacturer's instructions. After solubilization, extracted proteins (500 *μ*g) were first precleared by incubating lysates with sepharose beads for 1 h at 4°C and the supernatant was incubated overnight with the antibody at 4°C. Precipitation of the immune complexes was carried out for 2 h at 4°C and washed three times with the extraction buffer. Fractions were analyzed through standard SDS-PAGE and western blot techniques. To prevent protein deubiquitination during the experimental procedures, deubiquitinating enzyme inhibitors such as freshly made *N*-ethylmaleimide was added to all buffers

### Mitophagy measurement by flow cytometry

After treatments, cells were trypsinized for 5 min at 37°C, resuspended in complete medium with 10 nM Mito Tracker Deep Red (M22426, Invitrogen) and then incubated for 15 min at 37°C. For selected experiments, cells were preloaded with 10 nM of Mito Tracker Deep Red, incubated for 15 min at 37°C, washed and then treated with the different compounds. Using a LSRFortessa flow cytometer (Becton Dickinson) 20,000 cells were acquired (FACSDiva Software) and data analysed using the single cell analysis software FlowJo. Mean fluorescence channel in the FL4 channel in the viable cell population was plotted and normalized against that of untreated cells as described [22]. Mitophagy flux compares the MTDR levels with and without lysosomal inhibitors and is calculated as the as the ratio of MTDR fluorescence in the presence of lysosomal inhibitors to that in the absence of inhibitors, normalized to the corresponding value in control cells.

### Electron microscopy analysis

Cells were fixed using a solution of 2.5% glutaraldehyde. Cells were scrapped and centrifuged for 5 min. Cell pellets were post-fixed in osmium tetroxide, dehydrated through ascending concentrations of ethanol and embedded in epoxy resin. Ultra-thin sections were obtained at 0.1 μm, counterstained with uranyl acetate and lead citrate before observed by transmission electron microscopy (Hitachi-HU12A).

### Statistical analysis

Results are expressed as mean ± SEM. Differences between two groups have been analysed by an unpaired two-tailed Student's *t*-test. Differences between more than two groups were analysed by one-way analysis of variance (ANOVA) with Bonferroni's *post hoc* test for multiple comparisons. Statistical significance was set to *P* < 0.05.

## SUPPLEMENTARY MATERIALS FIGURES



## Abbreviations

AA: antimycin A, Apo, Apolipoprotein
Drp-1: dynamin-related protein 1
EGTA: ethylene glycol-bis(βamino-ethylether)-N, N, N', N',-tetraacetic acid
ER: endoplasmic reticulum
human VSMC: human vascular smooth muscle cell
LC3: microtubule-associated protein-1 light-chain 3
3-MA: (3-Methyladenine)
mitofusin 2: Mnf2
MTDR: Mito Tracker Deep Red
MFC: mitochondrial fragmentation count
MTT: 3-(4,5-dimethylthiazol-2-yl)-2,5-diphenyltetrazolium bromide
oxidized LDL: oxidized Low Density Lipoproteins
PI: Propidium Iodide
PINK1: PTEN-induced putative kinase 1
PS: phosphatidylserine
OMM: outer mitochondrial membrane
ROS: Reactive oxygen species
siRNA: Small interfering RNA
VDAC1: voltage-dependent anion channel 1
