# Supplementary material for: Mitophagy acts as a safeguard mechanism against human vascular smooth muscle cell apoptosis induced by atherogenic lipids
Source: Oncotarget. 2016 Apr 22;7(20):28821–35. doi: 10.18632/oncotarget.8936 (PMC5045359; doi:10.18632/oncotarget.8936)
Supplement: Supplementary file 1 [file oncotarget-07-28821-s001.pdf]

Mitophagy acts as a safeguard mechanism against human vascular smooth muscle cell apoptosis induced by atherogenic lipids

Supplementary Material

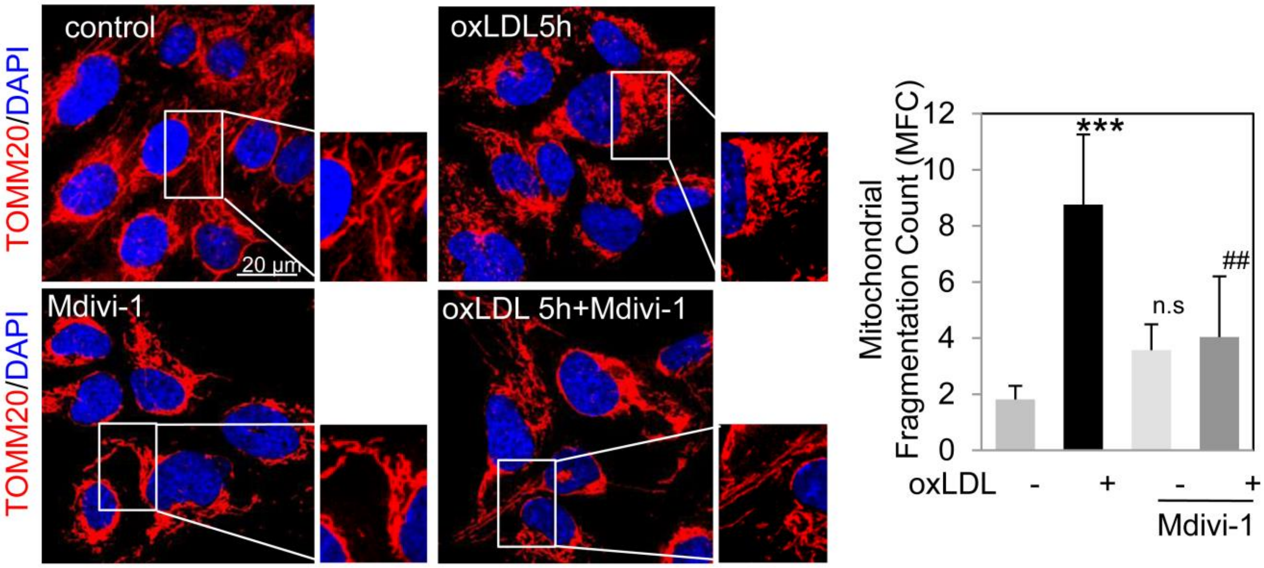

Figure S-I. The inhibitor of Drp-1 Mdivi-1 inhibits mitochondrial fission induced by oxidized LDL in human VSMC. Representative images of mitochondrial fragmentation/fission. Reversal of the mitochondrial fragmentation in human VSMC was achieved using Mdivi-1 inhibitor. Images are representative of human VSMC treated with oxidized LDL (200 µg ApoB/mL) for 5h, undergoing Mdivi-1 treatment. Mitochondria were stained using an antibody against the outer mitochondrial membrane-localized protein TOMM20 (red). Nuclei (blue) were stained with DAPI (4′,6-diamidino-2-phenylindole). The graph represents the quantification of the MFC and shows a significant reduction in oxidized LDL stimulated cells treated with the Dp-1 inhibitor Mdivi-1. Data are expressed as mean ± SEM of 3 separate experiments, *** $P < 0.001$ and ## $P < 0.01$ indicate significance, n.s indicates no significance.

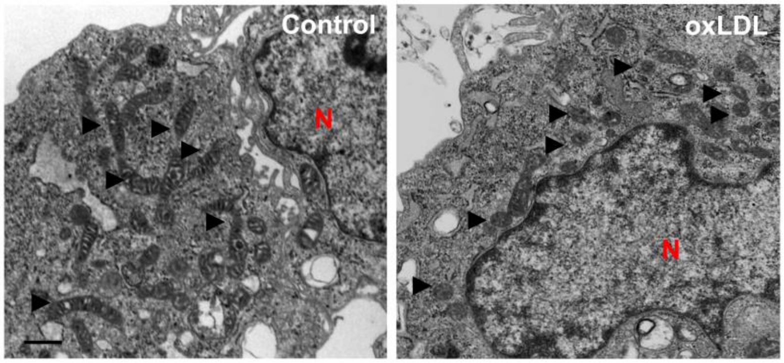

Figure S-II. Representative electron microscopy images of mitochondria from human VSMC after fixation and transmission electronic microscopy analysis. Control cells show bean-shaped structures with numerous transversely orientated cristae (note that the mitochondria (arrowheads ►) are distributed between the cytoplasm and around the nucleus (N). Human VSMC treated with oxidized LDL (200 µg ApoB/mL) show alterations in mitochondria morphology with small spherical mitochondria (arrowheads ►) that indicate fission events. Scale bar: 1µm

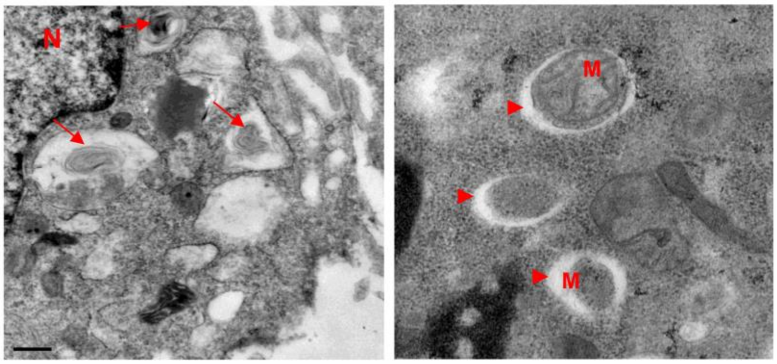

Figure S-III. Representative electron microscopy images of from human VSMC treated with oxidized LDL (200 µg ApoB/mL) showing features of autophagy and mitochondria engulfed in autophagy vesicles after fixation and transmission electronic microscopy analysis. (N), nucleus; (M) mitochondria; arrows (myeline figures); arrowheads (autophagy vesicles). Scale bar: 500 nm

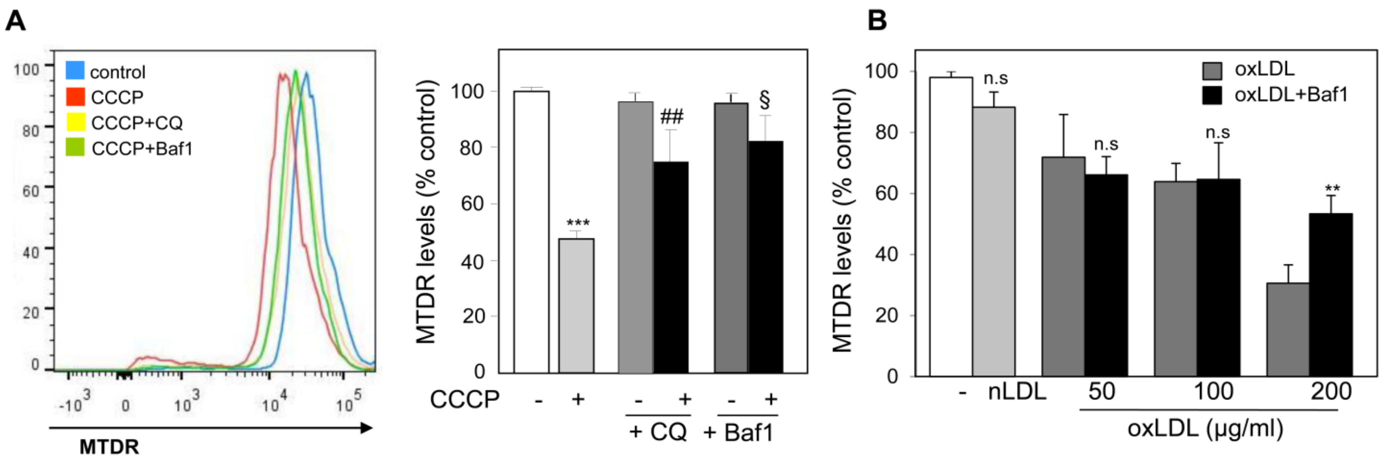

Figure S-IV. Flow cytometry analysis of mitophagy in human VSMC. (**A)** Cells were incubated with CCCP (20 µM) for 6 h and treated with or without Baf1 (10 nM) or CQ (10 µM) 3 h before analysis to block lysosomal degradation. Human VSMC were then stained with MTDR for flow cytometry analysis. The data are expressed as mean ± SEM of 4 separate experiments, *** *P*< 0.001, ## *P*< 0.01 and § *P*< 0.05 indicate significance. (**B)** Cells were incubated with native LDL and 50, 100 or 200 µg ApoB/mL of oxidized LDL for 8 h and treated with or without Baf1 (10 nM), then the cells were stained with MTDR for flow cytometry analysis. The data are expressed as mean ± SEM of 6 separate experiments, n.s indicates non significant and ** *P*< 0.01 indicates significance.

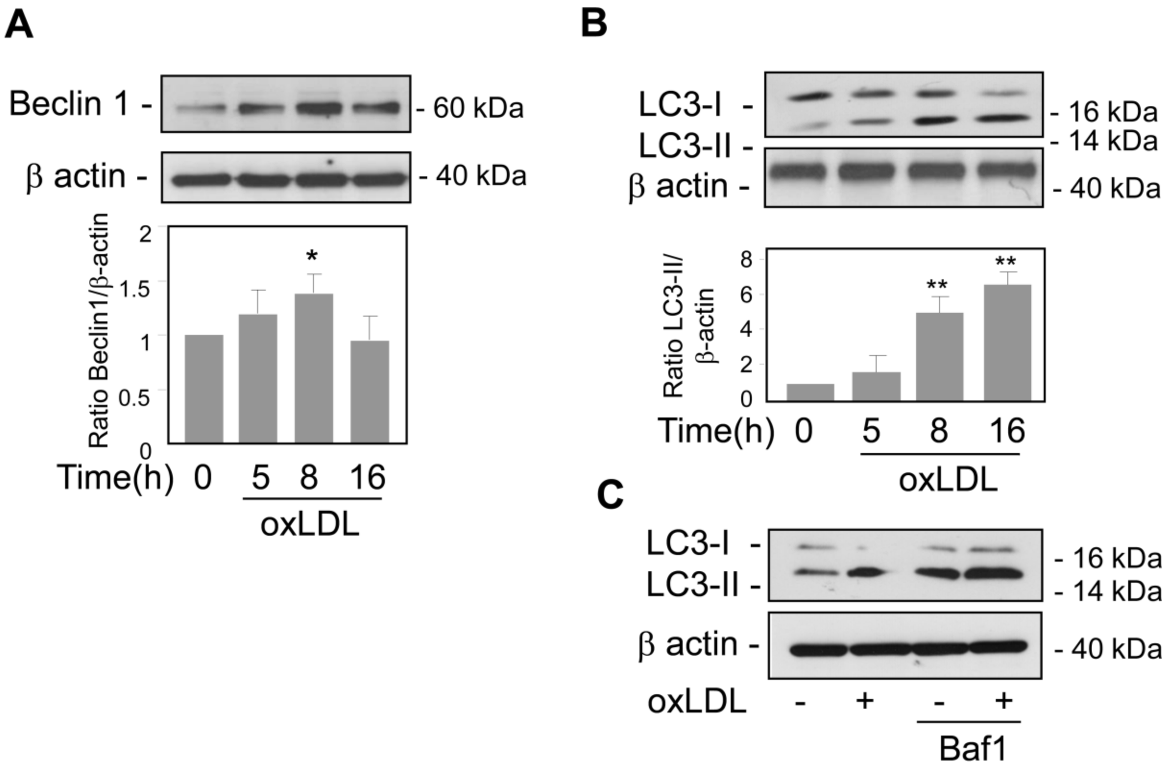

Figure S-V. Determination of autophagy machinery in oxidized LDL-stimulated human VSMC. (**A**) Immunoblot analysis of Beclin-1 expression following oxidized LDL treatment. Human VSMC were stimulated with oxidized LDL (200 µg ApoB/mL) at the indicated times and Western blot experiments were performed on total protein extracts using anti-Beclin 1 antibody and β-actin expression was used as loading control. Blots are representative of 3 independent experiments. The graph represents values (means ± SEM) of Beclin 1 band intensity after normalization for β-actin by densitometry, * $P< 0.05$ indicates significance. (**B**) Time course conversion of LC3-I to the phosphatidylethanolamine (PE)-conjugated form LC3-II in human VSMC cells treated by oxidized LDL (200 µg ApoB/mL) or (**C**) preincubated with Baf1 10 nM for 2 h. Western blot experiments were performed on total protein extracts using anti-LC3 antibody and β-actin expression was used as loading control. The graph represents values (means ± SEM) of LC3-II band intensity after normalization for β-actin by densitometry, ** $P< 0.01$ indicates significance

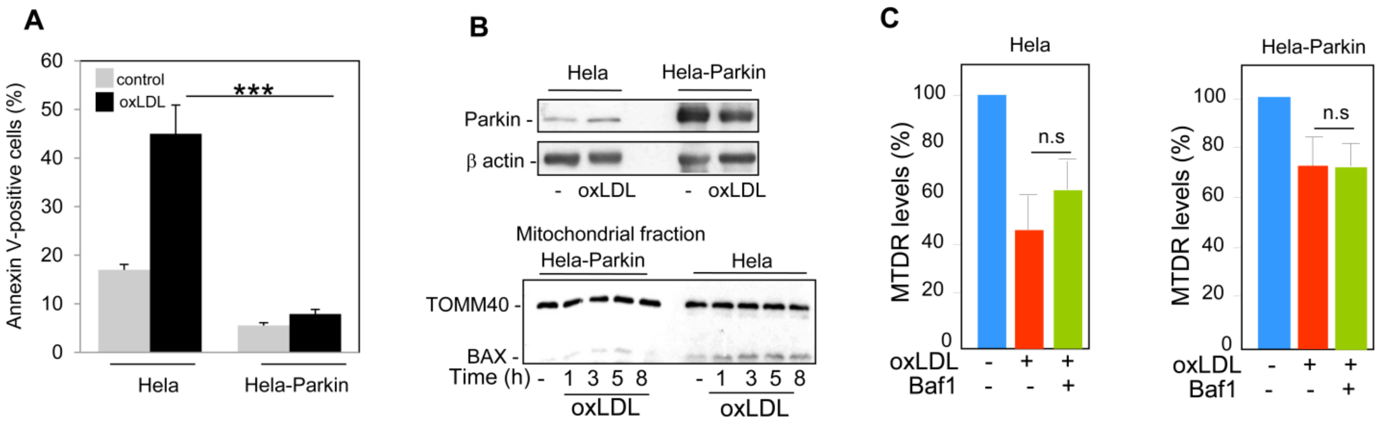

Figure S-VI. Parkin overexpression in Hela cells prevents the induction of apoptosis induced by oxidized LDL. (**A**) Apoptosis of Hela and Hela-Parkin cells stimulated with oxidized LDL (200 µg ApoB/mL) for 16 h was determined by Annexin V/PI staining followed by flow cytometry analysis. The graph represents the quantitative analysis of the percentage of Annexin V-positive cells. The data are expressed as mean ± SEM of 6 separate experiments, *** $P< 0.001$ indicates significance. (**B**) Expression of Parkin and Bax proteins in Hela and Hela-Parkin cells stimulated with oxidized LDL (200 µg ApoB/mL) for 16 h. Western blot experiments were performed on total protein extracts using anti-Parkin, anti-Bax, anti-TOMM40 and β-actin antibodies. The immunoblots are representative of 3 independent experiments. (**C**) Flow cytometry analysis of mitophagy in Hela and Hela-Parkin cells. Cells were incubated 200 µg ApoB/mL of oxidized LDL for 8 h and treated with or without Baf1 (10 nM) 3 h before analysis to block lysosomal degradation. Cells were then stained with MTDR for flow cytometry analysis. The data are expressed as mean ± SEM of 6 separate experiments. n.s indicates non significant .

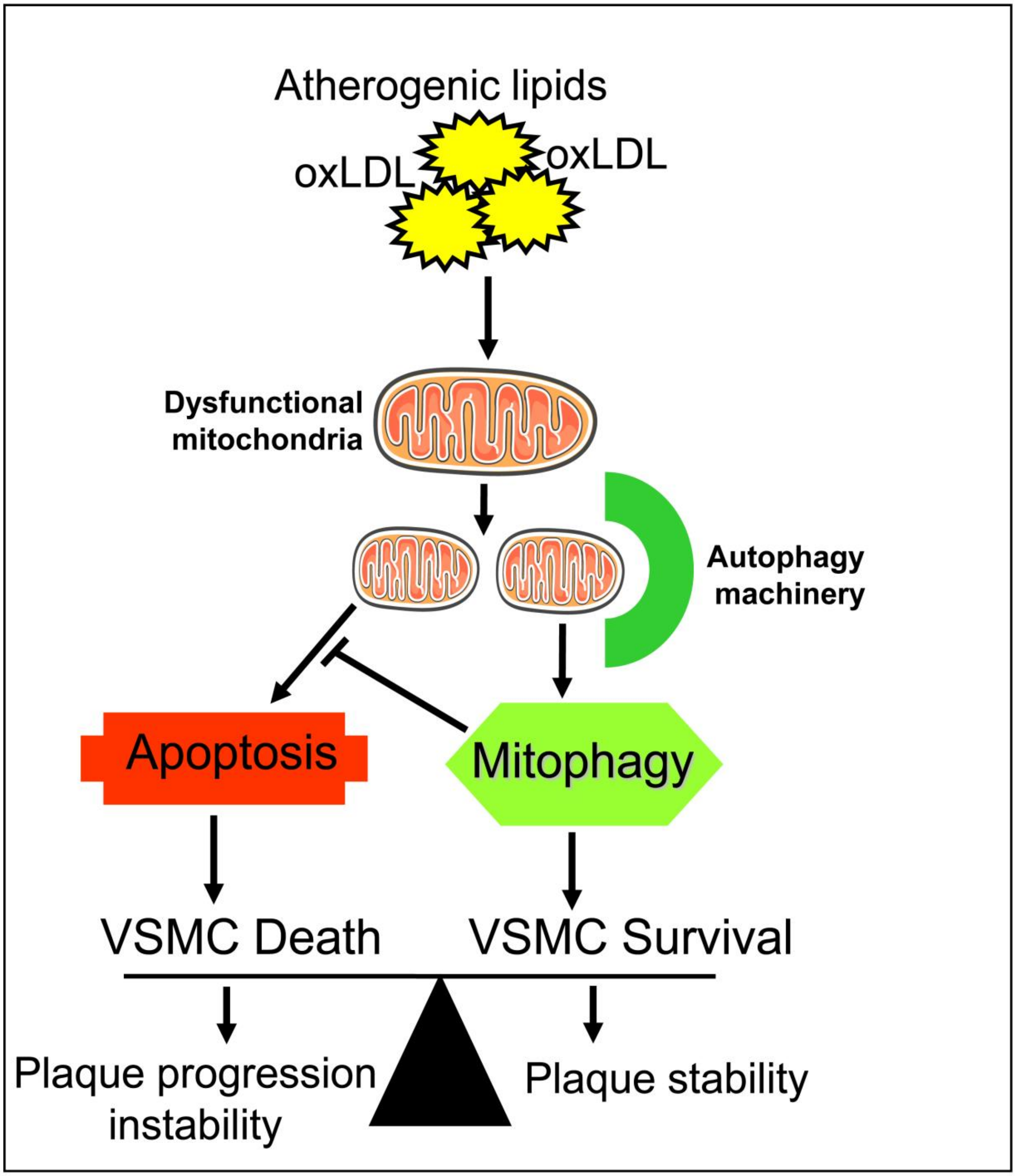

Figure S-VII. Summary scheme showing the coexisting effects of oxidized LDL on cell death and cell survival pathways in VSMC. In the current study we provided evidence for the occurrence of selective degradation of dysfunctional mitochondria through mitophagy to safeguard VSMC against oxidized LDL-induced apoptosis. This process takes on importance in the regulation of the cell survival/death balance and raises the interest to target selectively mitophagy to stabilize atherosclerotic plaque.
